# Plant-related Philistine ritual practices at biblical *Gath*

**DOI:** 10.1038/s41598-024-52974-9

**Published:** 2024-02-12

**Authors:** Suembikya Frumin, Aren M. Maeir, Maria Eniukhina, Amit Dagan, Ehud Weiss

**Affiliations:** 1 Archaeobotany Lab, The Martin (Szusz) Department of Land of Israel Studies and Archaeology, Bar-Ilan University, Ramat-Gan, Israel; 2 The Institute of Archaeology, Bar-Ilan University, Ramat-Gan, Israel; 3 The Martin (Szusz) Department of Land of Israel Studies and Archaeology, Bar-Ilan University, Ramat-Gan, Israel

**Keywords:** Plant sciences, Environmental sciences

## Abstract

The Philistine culture (Iron Age, ca. 1200-604 BCE) profoundly impacted the southern Levant's cultural history, agronomy, and dietary customs. Nevertheless, our knowledge of the Philistines’ cultic praxis and deities, is limited and uncertain. Here, we combine archaeological data with a meticulous study of plant use at two successive temples at Tell eṣ-Ṣâfī/Gath. We provide a list of the plants used, their time of harvest, mode of offering, and possible symbolism. Analysis of the temples' macrobotanical (seed and fruits) plant assemblage reveals the offerings; that the inception date for rites was early spring; and sheds light on the date of the final utilization of the temples (late summer/early fall). Besides food crops, we note the earliest cultic use of chaste tree (*Vitex agnus-castus*), crown daisy (*Glebionis coronaria*), and scabious (*Lomelosia argentea*). These wide-spread Mediterranean plants were known so far only in later cults—of early Greek deities, such as Hera, Artemis, Demeter, and Asclepios. We discuss the data as reflecting that the Philistine religion relied on the magic and power of nature, such as fresh water and seasonality, which influence human life, health, and activity. In sum, our results offer novel insights into the culture of the Philistines.

## Introduction

**Figure 1:**
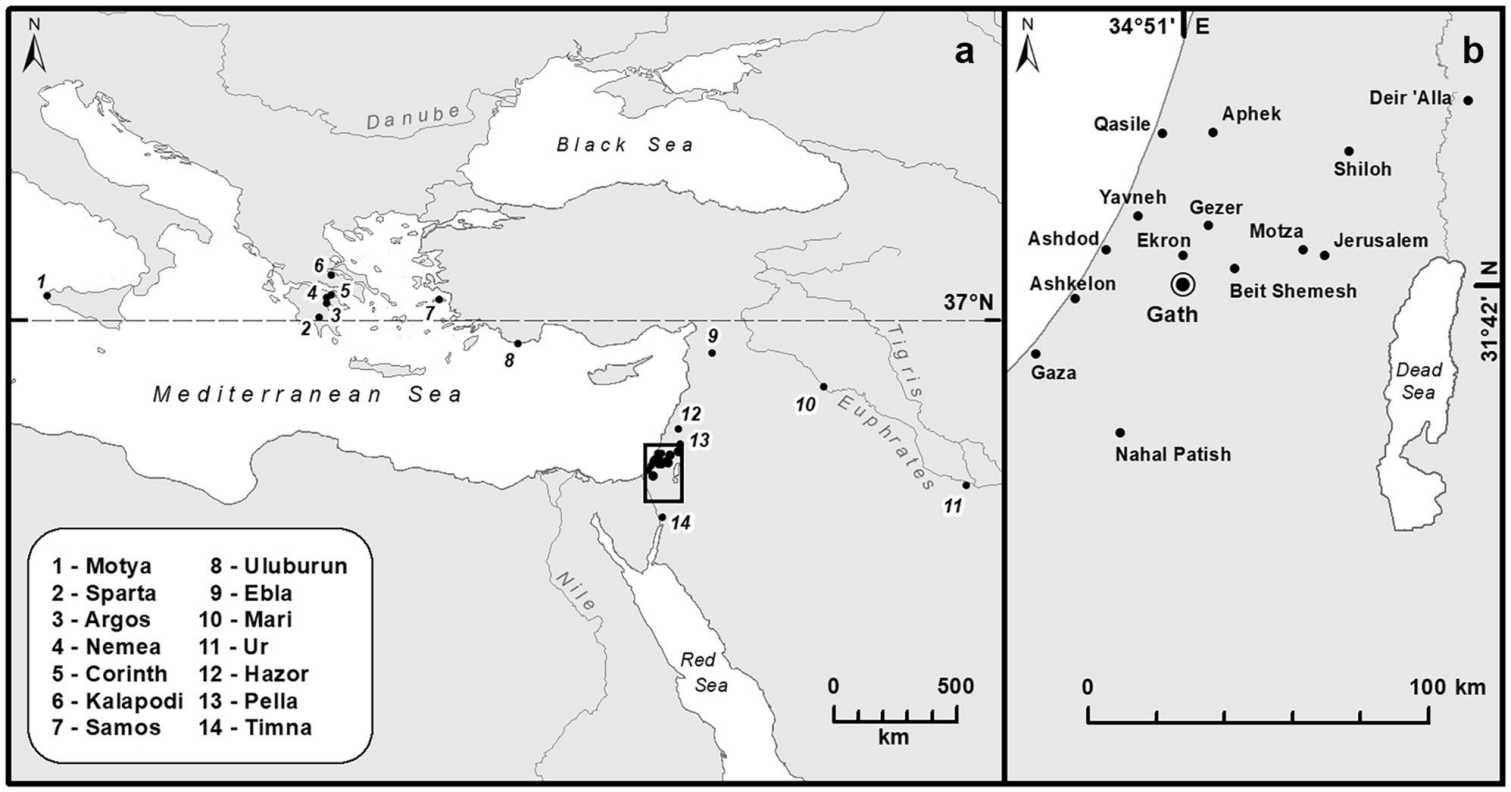
(**a**) Greater Near Eastern region, showing main archaeological sites mentioned in the text. (**b**) Southern Levant, showing location of the Tell eṣ-Ṣâfī/Gath (as Gath) and other sites mentioned in text. ArcGIS Desktop 10.6 https://www.esri.com/.

**Figure 2:**
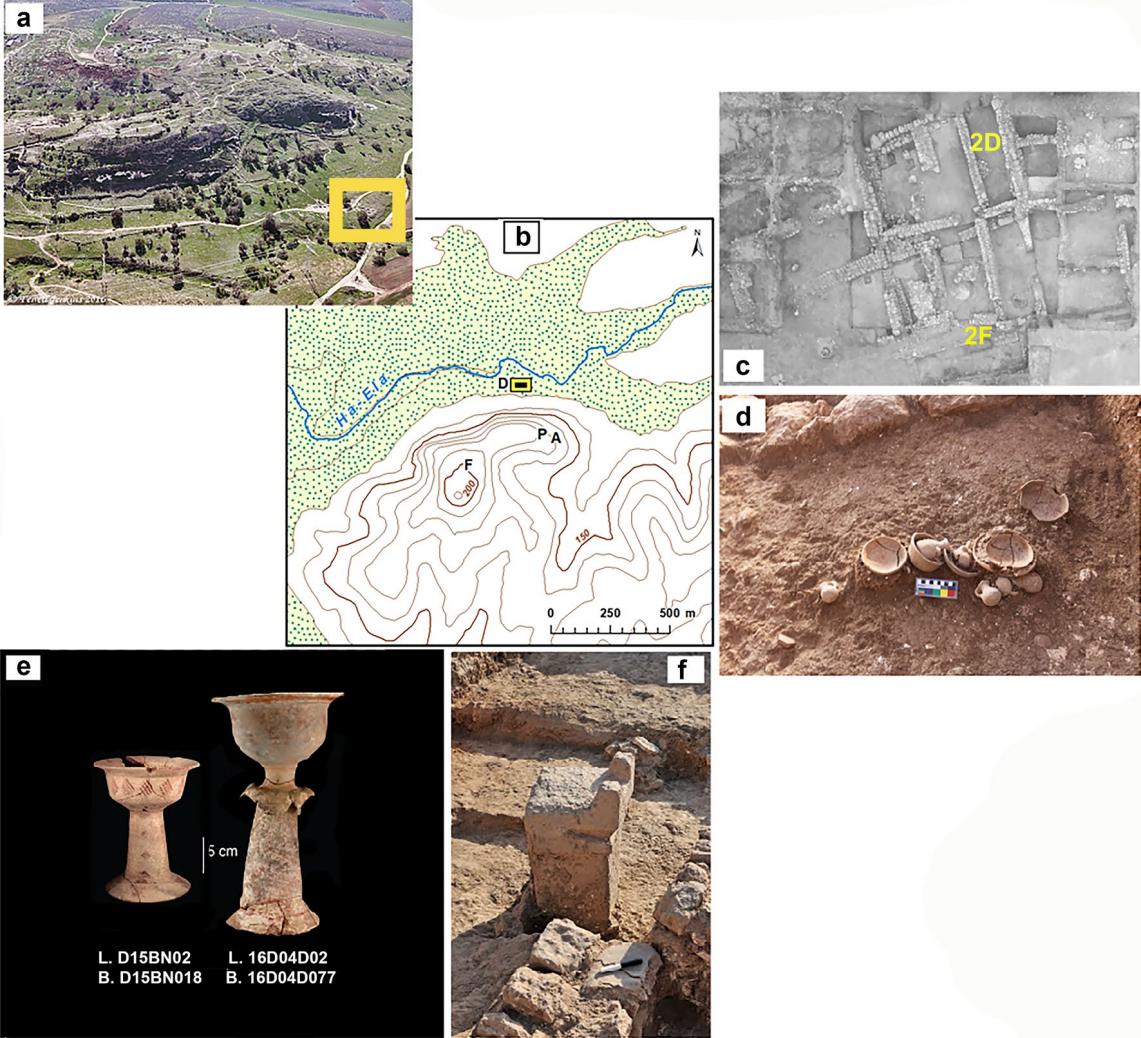
View of Tell eṣ-Ṣâfī/Gath showing the location of the study area (Area D) and votive offerings found in the temples. Photos 2a, 2c–f by A. Maeir. (**a**) View of northern side of Tell eṣ-Ṣâfī/Gath, showing Area D temples (yellow rectangle) and the valley to the north of the site. (**b**) Topographical map of Tell eṣ-Ṣâfī/Gath, showing location of excavation areas, the HaEla riverbed course and the valley to the north of the site. The yellow rectangle marks the temples’ location. ArcGIS Desktop 10.6 https://www.esri.com/. (**c**) Aerial photo of Area D with temples and surrounding buildings. Yellow marks denote the location of offerings (**d**) and the altar (**f**). (**d**) Cultic assemblage of miniature vessels and an endolium shell (*Tonna galea*) found in temple D4. (**e**) Decorated chalices found in temple D3. (**f**) Altar found in temple D3.

**Figure 3:**
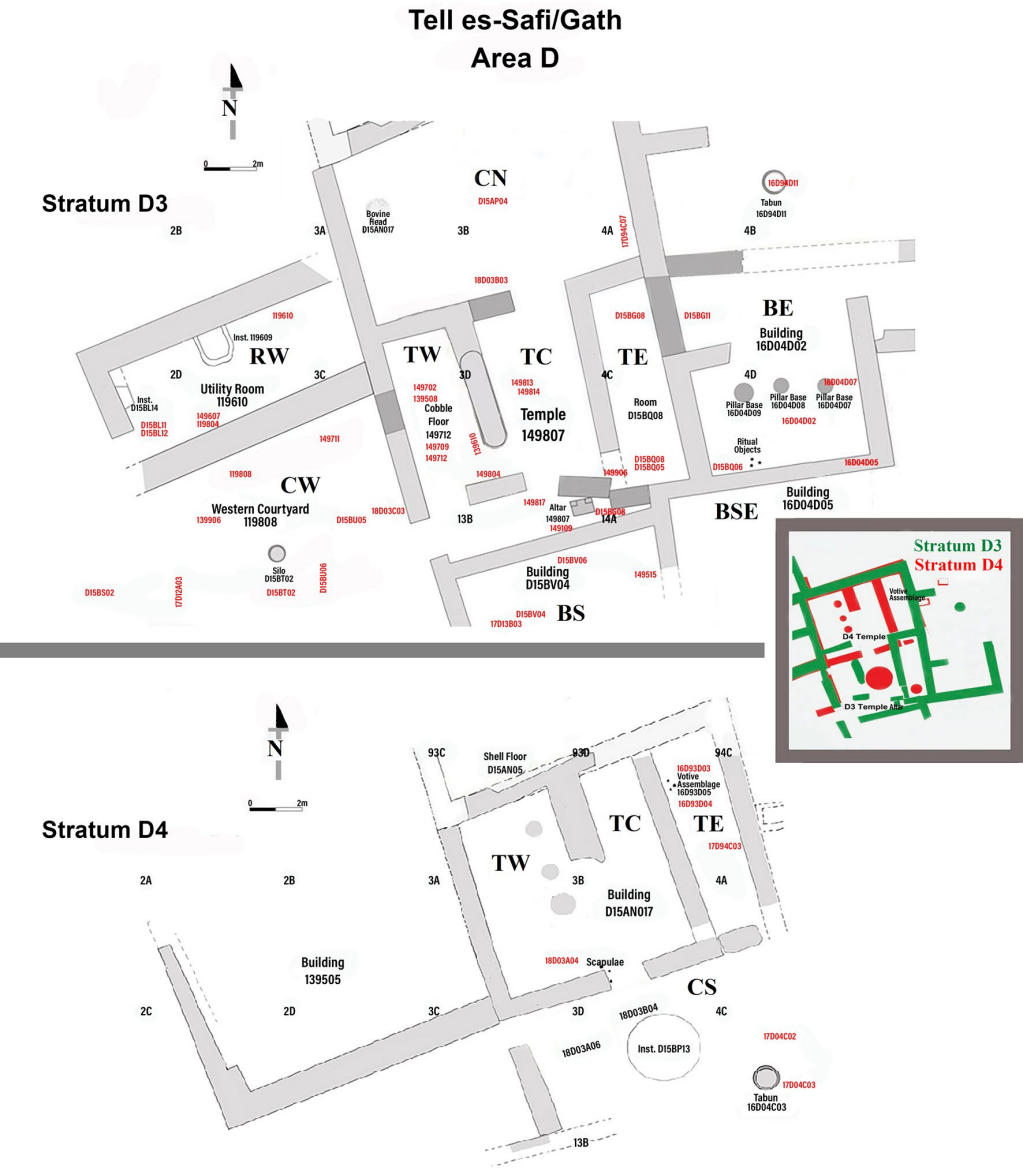
Top plan of Area D with delineation of the two temples: Stratum D4 and Stratum D3. Red color denotes loci sampled for archaeobotany. Letters mark type of space (Temple, Room, Courtyard, Building) and their orientation (N, E, S, W). Ex: CN – northern courtyard; TC – temple central room; RW – western room; BE – eastern building).

Excavation at the largest Philistine settlement, Tell eṣ-Ṣâfī/Gath (hitherto Gath), identified as biblical *Gath of the Philistines* and the home of Goliath (Figs. 1, 2, 3), exposed two successive temples in the lower city, on the banks of HaEla river (Area D)^1^. There, archaeobotanical sampling yielded an impressive assemblage of seeds and fruits^2^. The qualitative and quantitative study of the assemblage and its spatial distribution within the temples' precincts enables us to address plant choice and possible use, unraveling the timing of rites, types of offers, and improving our understanding of this extinct culture. Indeed, plants in ritual contexts shed light on the seasonality of rites, the role of agriculture, medical/psychoactive activities, and the geographic origin of offerings^3–9^.

Fresh water, agriculture, and the cyclical birth, death, and rebirth of a plant are recognized and venerated as transformative, and even magical, in the oldest myths, such as the Gilgamesh epic, the tale of Aqhat, and the worship of deities such as Tammuz, Ishtar, and Baal^10–14^. There is evidence of cultural connections between specific cultic traditions and certain plants, such as Demeter and barley (*Hordeum vulgare*), and Late Bronze Age Egyptian rites related to the white lotus (*Nymphaea lotus*).

**Figure 4:**
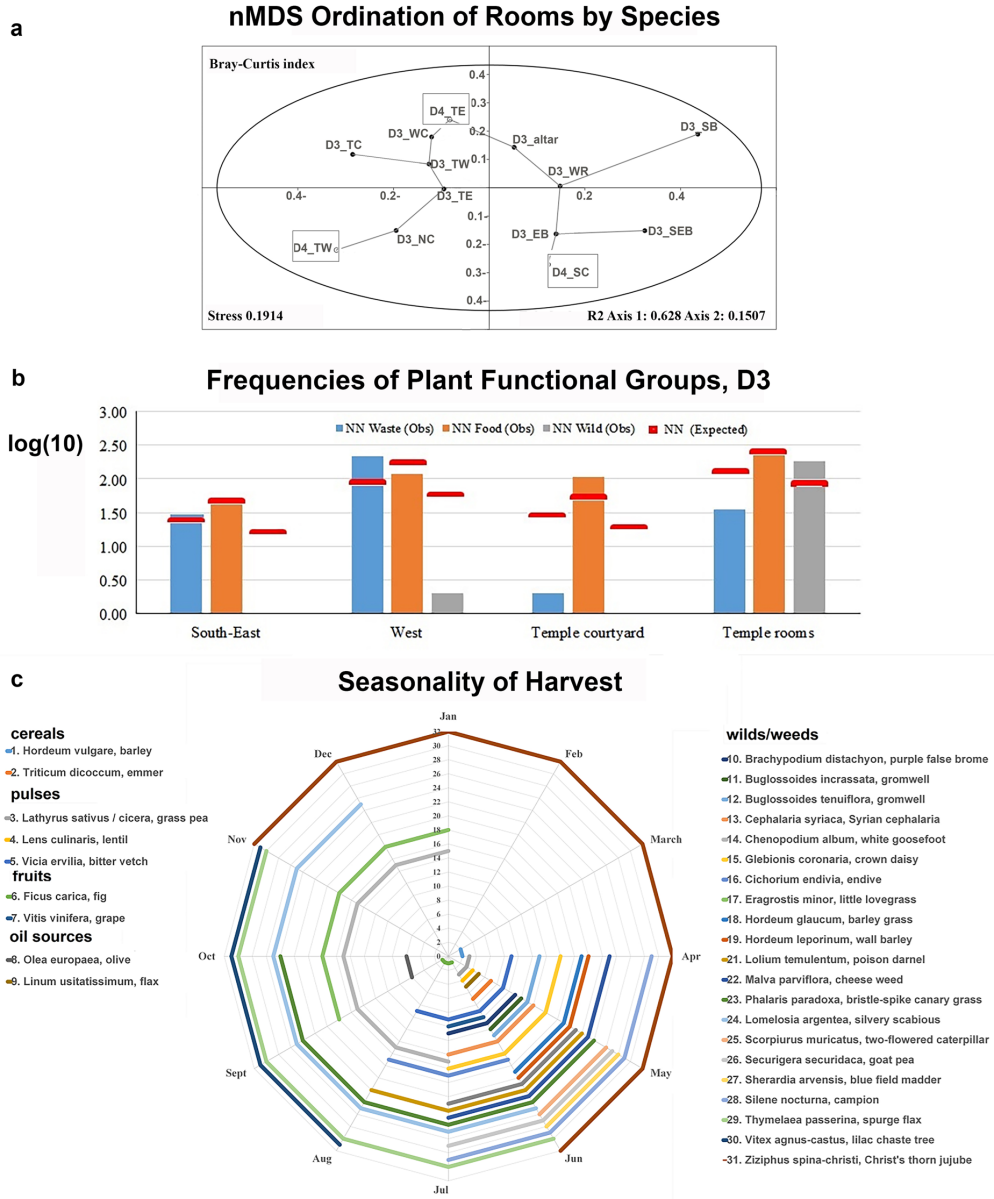

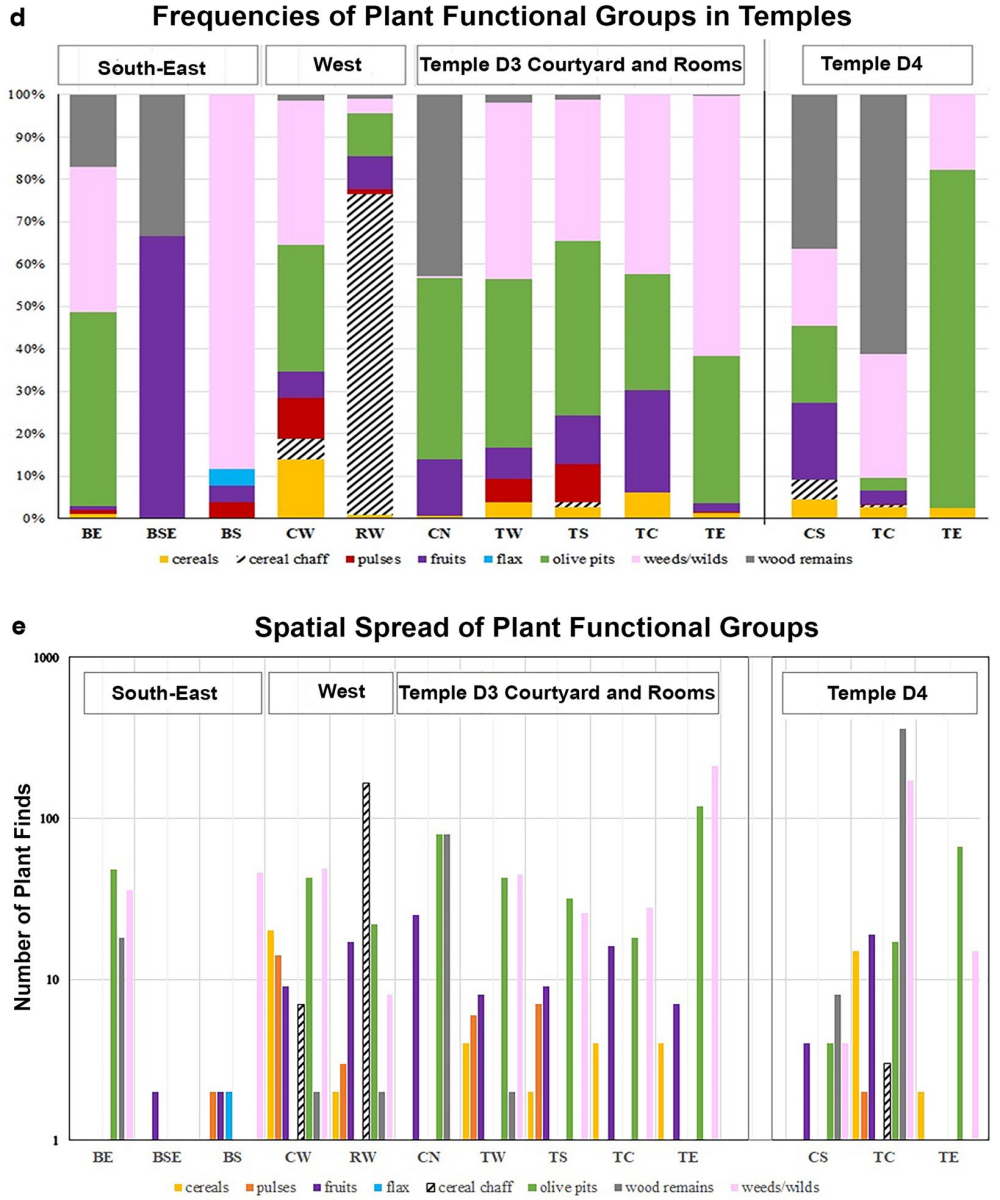
Plants spread inside and around the temples’ precincts. (**a**) Non-metric nMDS ordination test by the Bray–Curtis similarity index between the temples’ spaces by species composition. Each location is marked in the following order: Strata (D3 or D4), orientation in relation to the temple center (N, W, S, E), and type of space (altar; R—room, C—courtyard, B—building). (**b**) Frequencies of Plant Functional Groups within the Temple D3 (log-transformed for visibility). Colored bars represent observed frequencies of plant functional groups; Red bar – the expected frequency. (**c**) Seasonality of Plants’ Harvest. Plants are numbered and arranged according to their belonging to the functional group (crops, weed/wilds). (**d**) Frequencies of plant functional groups in the temples’ D3 and D4 areas. (**e**) Spatial spread of plant functional groups in the D3 and D4 Temple areas.

**Figure 5:**
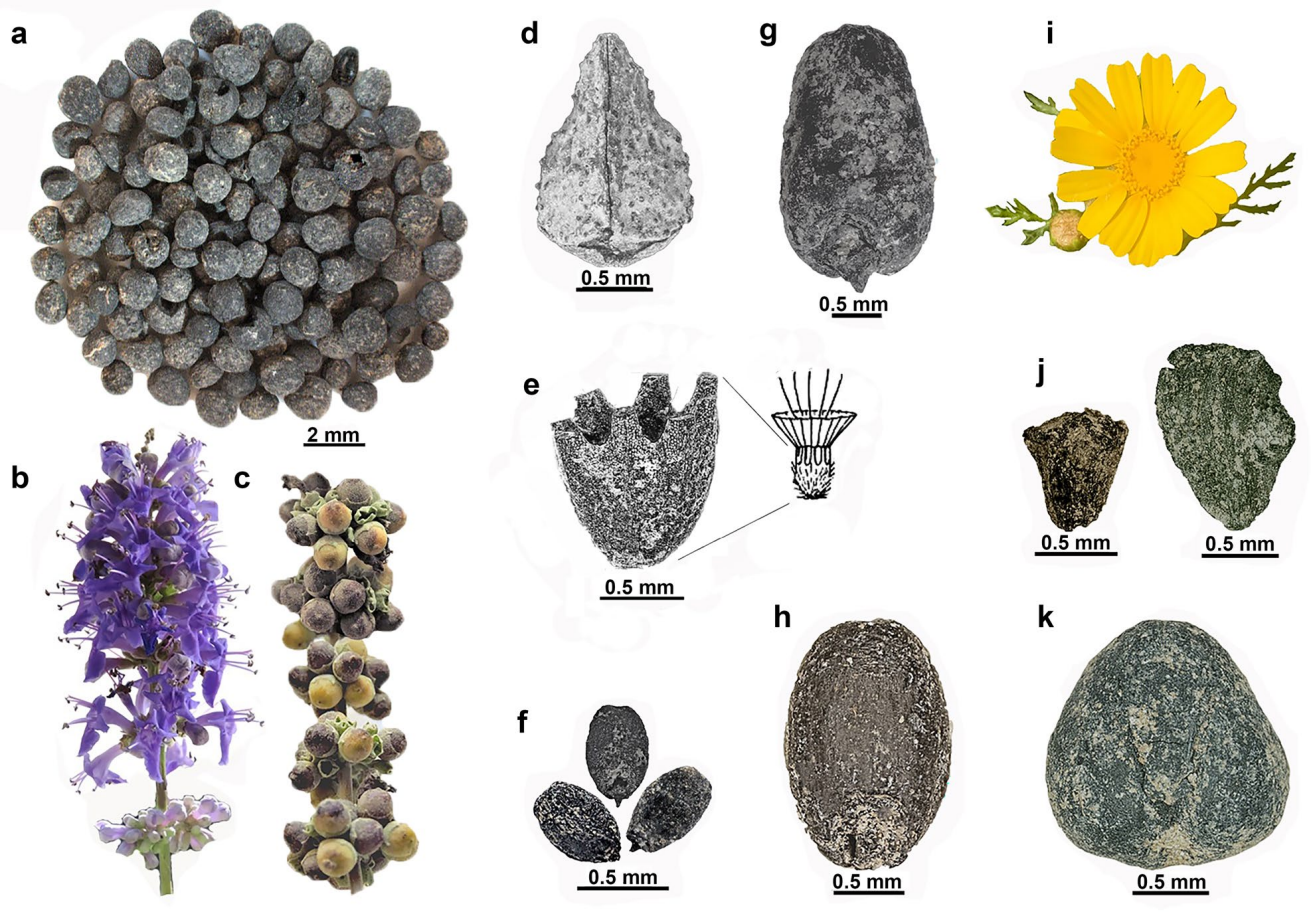
Plants of the Philistine temples at Tell eṣ-Ṣâfī/Gath. All Photos by S. Frumin. Seed photographs made using stereoscopic light microscope Olympus SZ×10 DP73 and digital scanning using cellSens Dimension 1.9 program, Adobe Photoshop 2024 was used for background editing. (**a–c**) *Vitex agnus-castus*, chaste tree. (**a**) Chaste tree fruits found in D3 (L 149813). (**b**) Chaste tree, flowering modern inflorescence. (**c**) Chaste tree, modern fruiting branch. (**d**) *Buglossoides incrassata*, gromwell, nutlet (L D15BW08). (**e**) *Lomelosia argentea*, silvery scabious, persistent outermost whorl of inflorescence (calyx) – the archaeological find from L 16D93D03 and schematic drawing of the complete calyx. (**f**) *Eragrostis minor*, little lovegrass, grains (L 149515). (**g**) *Triticum parvicoccum*, free-threshing wheat grain (L 149515). (**h**) *Lolium temulentum*, poison darnel, grain (L 16D93D03). (**i**) *Glebionis coronaria*, crown daisy, flowering head (inflorescence, modern). (**j**) *G. coronaria* fruits (achenes) found in the temples (L 16D93D03). (**k**) *Vicia ervilia,* bitter vetch, seed (L 16D93D03).

Although a plant’s role may change with time and across biogeographic regions, it is plausible to assume that connected cultures and peoples shared knowledge of natural resource use and cultural appreciation of landscape components. Indeed, the famous physician Dioscorides (41–68 CE) provides a collection of synonyms for the lilac chaste tree (*Vitex agnus-castus*, Fig. 5a–c), indicating its widespread use in various ancient Mediterranean cultures (Fig. 1a). Chaste tree was used at the female agricultural festival of Thesmophoria, celebrated in Greek cities to honor the goddesses Demeter and Persephone. The plant was known as a medicinal and was used as an abortifacient. In Archaic Sparta (eighth-sixth centuries BCE), the plant was used in the cult of the local goddess of agriculture—Artemis Orthia (*Lugodesma*)(^15^:16.9–11). Pausanias (second century CE) noted that the wooden image of Asclepios (*Agnitas*) at Sparta was made from the chaste tree (^15^:2.35.4–8). There is also botanical and textual evidence for the significance of the chaste tree in the Heraion on Samos during the sixth-third centuries BCE^16,17^.

The entangled Philistine Iron Age Levantine culture (ca. 1200–600 BCE) combined elements of Aegean, Egyptian, Canaanite, and other cultures^18,19^. While biblical depictions present a polytheistic Philistine culture worshipping gods and goddesses, the details and exact identities of their deities remain largely unknown^20^. Despite intensive research on Philistine culture, relatively little is known about their cultic practices, including the use of plants in ritual contexts and the role of temples for communal crop storage and public feasting^21,22^. The only known plants from a Philistine temple, Tell Qasile (mid-eleventh century BCE, Fig. 1b), are seed caches dominated by Aegean staple pulses^3^. Noteworthy, Philistine plant use in non-cultic contexts also combined local crops with the addition of novelties in vegetables, condiments, agronomy, and processing^2,23–25^. Most intriguing was the presence of local wild useful plants (e.g., bay tree, coriander, henbane), that are unknown in Israeli archaeobotanical data for the earlier Bronze Age^25^. Unfortunately, these data shed no light on the possible connections to specific rites and deities.

The plant assemblage originated from two temples built one on top of another: the earlier in Stratum D4 (ca. tenth century BCE; hitherto D4) and the later in Stratum D3 (ending ca. 830 BCE; hitherto D3; see Table 1, Fig. 3, and Suppl. Table). The final destruction of the temple is associated with the conquest of the city by King Hazael of Aram-Damascus. It should be stressed that both in the Stratum D3 Temple and in other parts of the city, a thick and largely undisturbed destruction level was revealed (ca. 830 BCE), and the finds discussed here come from clearly undisturbed contexts relating to this destruction (without later disturbances)^26,27^. The studied temples include inner rooms with rich votives, and numerous loom weights as evidence of weaving at the temples; adjacent courtyards with cooking facilities, and a metallurgic industrial zone in the immediate vicinity (Figs. 2, 3)^28,29^. Noteworthy, near the altar in the temple (L 149107), a storage jar produced in the Jerusalem area was found, which could be used for food storage and transportation. This indicates that some of the final offerings originated from the region of Judah^30^.

**Table 1:** Seeds and fruits assemblage from the temples in Gath.

	Stratum	Stratum D3											Stratum D4			
	Locus code	BE	BSE	BS	CW	RW	Temple, CN	Temple, RW	Temple, RS	Temple, RC	Temple, RE	D3, Total	Temple, CS	Temple, RE	Temple, RC	D4, Total
	Locus #	BUILD. 16D04D02	BUILD. 16D04D05	BUILD. D15BV04	COURT. 119,808	ROOM 119,610	COURT. D15AP04	ROOM 149,702	ROOM 149,807	ROOM 149,814	ROOM D15BQ08		COURT. D15BP13	ROOM 16D93D05	ROOM D15AN07	
	Sediment Volume, V (L)	164	24	85	118	27	37	73	158	110	145	***941***	65	371	39	***475***
	Number of plant finds, N	105	3	52	141	162	187	107	82	213	226	***1272***	45	564	84	***693***
	Density = N/V	0.64	0.13	0.61	1.19	6.00	5.05	1.47	0.52	1.94	1.56	***AV 1.9***	0.69	1.52	2.15	***AV 1.5***
Cereals	Organ	Count (% per column)										**Sum for D3 (%)**	Count (%)			**Sum for D4 (%)**
*Hordeum vulgare*	Grain	1 (1%)			3 (2%)							***4 (*<** ***1%)***	1 (2%)	3 (< 1%)		***4 (*<** ***1%)***
*Triticum dicoccum*	Grain				7 (5%)			1 (1%)		2 (1%)		***10 (1%)***		2 (< 1%)		***2 (*<** ***1%)***
	Glume base					104 (64%)			1 (1%)			***105 (9%)***				
*Triticum dicoccum,* cf.	Grain										1 (< 1%)	***1 (*<** ***1%)***		2 (< 1%)		***2 (*<** ***1%)***
*Triticum parvicoccum*	Grain				7 (5%)		1 (< 1%)	2 (2%)	2 (2%)	1 (< 1%)	2 (1%)	***15 (1%)***		8 (1%)	2 (2%)	***10 (*>** ***1%)***
	Axis frgmnt					2 (1%)						***2 (*<** ***1%)***				
*Triticum parvicoccum,* cf.	Grain										1 (< 1%)	***1 (*<** ***1%)***				
*Triticum* sp.	Grain				1 + 2f. (2%)	2f. (1%)				1 (< 1%)		***1*** **+** ***4f. (*<** ***1%)***		3f. (< 1%)		***3f. (*<** ***1%)***
*Triticum/Hordeum*	Grain							1f. (1%)				***1f. (*<** ***1%)***				
***Sum for Cereals: counts (% per column)***		***1 (1%)***	***0***	***0***	***18*** + ***2f. (14%)***	***106*** **+** ***2f. (67%)***	***1 (*<** ***1%)***	***4 (4%)***	***3 (4%)***	***4 (2%)***	***4 (2%)***	***139*** **+** ***5f. (11%)***	***1 (2%)***	***7*** **+** ***3f. (*<** ***1%)***	***2 (2%)***	***20 (*<** ***1%)***
Pulses		Count (% per column)										**Sum for D3 (%)**	Count (%)			**Sum for D4 (%)**
*Lathyrus sativus / cicera*	Seed				1 (< 1%)							***1 (*<** ***1%)***				
*Lens culinaris*	Seed	1 (1%)			5 (4%)			5 (5%)	3 (4%)		2 (1%)	***16 (*>** ***1%)***			2f. (2%)	***2f. (*<** ***1%)***
*Lens culinaris,* cf.	Seed													1 (< 1%)		***1 (*<** ***1%)***
*Lens,* cf.	Seed				1 (< 1%)	3 (2%)						***4 (*<** ***1%)***				
*Vicia ervilia*	Seed				7 (5%)				4 (5%)			***11 (1%)***		1 (< 1%)		***1 (*<** ***1%)***
*Vicia ervilia,* cf.	Seed							1 (1%)	1 (1%)			***2 (*<** ***1%)***				
*Vicia sp.*	Seed										2 (1%)	***2 (*<** ***1%)***		5f. (1%)		***5f. (*<** ***1%)***
*Vicia,* cf.	Seed	1 (1%)										***1 (*<** ***1%)***				
***Sum for Pulses: Counts (%)***		***2 (2%)***	***0***	***0***	***14 (10%)***	***3 (2%)***	***0***	***6 (6%)***	***8 (10%)***	***0***	***4 (2%)***	***37 (3%)***	***0***	***2*** **+** ***5f. (1%)***	***2f. (2%)***	***2*** **+** ***7f. (1%)***
Fruits		Count (% per column)										**Sum for D3 (%)**	Count (%)			**Sum for D4 (%)**
*Ficus carica*	Druplet				1 (< 1%)	1 (< 1%)	25 (13%)	4 (4%)		13 (6%)	4 (2%)	***48 (4%)***		4 (1%)		***4 (*<** ***1%)***
*Vitis vinifera*	Pip	1 (1%)	2 (67%)	2 (4%)	8 (6%)	6 + 10f. (10%)		4 (4%)	6 + 4f. (12%)	1 (< 1%)	4 (2%)	***34*** **+** ***14f. (4%)***	3 (7%)	11 + 4f. (3%)		***14*** **+** ***4f. (3%)***
***Sum for Fruits: Counts (%)***		***1 (1%)***	***2 (67%)***	***2 (4%)***	***9 (7%)***	***7*** **+** ***10f.***	***25 (13%)***	***8 (7%)***	***6*** + ***4f. (12%)***	***14 (7%)***	***8 (4%)***	***82*** **+** ***14f. (8%)***	***3 (7%)***	***15*** + ***4f. (3%)***	***0***	***18*** + ***4f. (3%)***
Oil plants		Count (% per column)										**Sum for D3 (%)**	Count (%)			**Sum for D4 (%)**
*Linum usitatissimum*	Seed			2 (4%)								***2 (*<** ***1%)***				
*Linum usitatissimum, cf.*	Seed								1 (1%)			***1 (*<** ***1%)***				
*Olea europaea*	Fruit stone	48f. (46%)			43f. (32%)	23f. (14%)	80f. (43%)	43f. (40%)	2w + 30f. (39%)	18f. (8%)	119f. (53%)	***2w*** **+** ***404f. (32%)***	4f. (9%)	17f. (3%)	67f. (80%)	***99f. (14%)***
***Sum for Fruits: Counts (%)***		***48f. (46%)***	***0***	***2 (4%)***	***43f. (32%)***	***23f. (14%)***	***80f. (43%)***	***43f. (40%)***	***3*** **+** ***30f.***	***18f. (8%)***	***119f. (53%)***	***4*** **+** ***404f. (33%)***	***4f. (9%)***	***17f. (3%)***	***67f. (80%)***	***99f. (14%)***
Weeds/ Wilds		Count (% per column)										**Sum for D3 (%)**	Count (%)			**Sum for D4 (%)**
*Achillea aleppica,* cf.	Achene													1 (< 1%)		***1 (*<** ***1%)***
*Achillea aleppica/ Cichorium endivia*	Cypsela of outside whorl										1 (< 1%)	***1 (*<** ***1%)***				
*Anagallis arvensis*	Seed										1 (< 1%)	***1 (*<** ***1%)***				
[U 64] *Apiaceae/ Euphorbiaceae*	Capsule														1 (1%)	***1 (*<** ***1%)***
[U 64] *Apiaceae, Cicuta* type	Achene													1 (< 1%)		***1 (*<** ***1%)***
*Brachypodium distachyon*	Grain													1 (< 1%)		***1 (*<** ***1%)***
*Brachypodium,* cf.	Grain													6f. (1%)		***6f. (1%)***
*Buglossoides incrassata*	Achene								1 (1%)			***1 (*<** ***1%)***				
*Buglossoides tenuiflora*	Achene							1 (1%)				***1 (*<** ***1%)***				
[U 63] *Cephalaria syriaca*	Fruit								1f. (1%)			***1f. (*<** ***1%)***				
[U 63] *Chenopodium album*	Seed													11 (2%)		***11 (2%)***
[U 63] *Chenopodium* cf*. album*	Seed							4 (4%)				***4 (*<** ***1%)***				
[U 63] *Chenopodium* sp.				3 (6%)	3 (2%)			3 (3%)				***9 (1%)***		16 (3%)		***16 (2%)***
[U 13] *Chrysanthemum coronarium*	Cypsela									2 (1%)		***2 (*<** ***1%)***		2 (< 1%)		***2 (*<** ***1%)***
[U 13] *Chrysanthemum coronarium,* cf.	Achene													3 (< 1%)		***3 (*<** ***1%)***
*Compositae*	Achene				1 (< 1%)							***1 (*<** ***1%)***				
[U 48] *Eragrostis minor*	Grain			22 (42%)								***22 (2%)***				
*Euphorbia helioscopia*	Fruit													1 (< 1%)		***1 (*<** ***1%)***
*Galium* sp.	Mericarp				1 (< 1%)						1 (< 1%)	***2 (*<** ***1%)***		1 (< 1%)		***1 (*<** ***1%)***
*Hordeum glaucum*	Grain				2 (1.5%)							***2 (*<** ***1%)***				
*Hordeum leporinum*	Grain				1 (< 1%)							***1 (*<** ***1%)***				
*Hordeum* sp.	Grain					1f. (< 1%)		1f. (1%)			2f. (1%)	***4f. (*<** ***1%)***				
*Liliaceae (Morea* type*)*	Seed													1 (< 1%)		***1 (*<** ***1%)***
*Lolium rigidum*	Grain														1 (1%)	***1 (*<** ***1%)***
*Lolium rigidum*, cf.	Grain							1 (1%)				***1 (*<** ***1%)***				
*Lolium* sp.	Grain	1 (1%)			1 (< 1%)	1 + 2f. (2%)					1 (< 1%)	***4*** **+** ***2f. (*<** ***1%)***				
[U 43, 44] *Lolium temulentum*	Grain				13 (10%)							***13 (1%)***		3 (< 1%)		***3 (*<** ***1%)***
[U 43, 44] *Lolium temulentum*, cf.	Grain													1 (< 1%)		***1 (*<** ***1%)***
*Lomelosia argentea*	Fruit base													1 (< 1%)		***1 (*<** ***1%)***
[U 64] *Malva parviflora*	Seed								1 (1%)			***1 (*<** ***1%)***				
	Gynophore													1 (< 1%)		***1 (*<** ***1%)***
*Malva* sp.	Seed										1 (< 1%)	***1 (*<** ***1%)***				
*Malvaceae*	Seed										2 (1%)	***2 (*<** ***1%)***				
*Medicago* sp.	Seed				1 (< 1%)							***1 (*<** ***1%)***				
*Papilionaceae*	Seed				1 (< 1%)			7 (6.5%)			1 (< 1%)	***9 (1%)***		2 (< 1%)		***2 (*<** ***1%)***
[U 64] *Papilionaceae,* large	Seed			1f. (2%)								***1f. (*<** ***1%)***				
[U 64] *Papilionaceae,* small	Seed									1 (< 1%)	22 (10%)	***23 (2%)***				
*Phalaris paradoxa*	Grain			3 (6%)	4 (3%)	1 (< 1%)		2 (2%)				***10 (1%)***		6 (1%)		***6 (1%)***
*Phalaris paradoxa,* cf.	Grain								1 (1%)			***1 (*<** ***1%)***				
*Phalaris* sp.	Grain				2 (1.5%)						3 (1%)	***5 (*<** ***1%)***		9 (2%)		***9 (*>** ***1%)***
*Poaceae*	Grain				2 (1.5%)			6 (6%)	2 (2%)			***10 (1%)***		1 (< 1%)		***1 (*<** ***1%)***
*Poaceae,* small-grained	Grain													8 + 5f. (2%)		***8*** + ***5f. (2%)***
[U 64] *Rumex* sp.	Nutlet													1 < 1%)		***1 (*<** ***1%)***
[U 51] *Scorpiurus muricatus*	Seed				3 (2%)		1 (< 1%)	3 (3%)				***7 (*<** ***1%)***				
[U 51] *Scorpiurus muricatus,* cf.	Seed														1 (1%)	***1 (*<** ***1%)***
*Securigera securidaca*	Seed				1 (< 1%)							***1 (*<** ***1%)***				
[U 48] *Sherardia arvensis*	Seed				1 (< 1%)							***1 (*<** ***1%)***				
*Silene nocturna*	Seed			1 (2%)								***1 (*<** ***1%)***				
*Thymelaea passerina*	Seed				2 (1.5%)			1 (1%)		1 (< 1%)		***4 (*<** ***1%)***				
*Vicia, smaller,* cf.	Seed	1 (1%)										***1 (*<** ***1%)***				
*Vicieae*	Seed			2 (4%)								***2 (*<** ***1%)***				
[U 13, 55, 56]* Vitex agnus-castus*	Fruit									171 (80%)		***171 (13%)***				
[U 64] *Ziziphus spina-christi*	Whole fruit stone												1 (2%)			***1 (*<** ***1%)***
***Sum for Wilds: Counts (%)***		***2 (2%)***	***0***	***31*** + ***1f. (62%)***	***39 (29%)***	***2*** + ***3f. (3%)***	***1 (***< ***1%)***	***28*** + ***1f. (27%)***	***5*** + ***1f. (7%)***	***175 (82%)***	***33*** **+** ***2f. (15%)***	***316*** + ***8f. (25%)***	***1 (2%)***	***71*** **+** ***11f. (14%)***	***3 (3%)***	***74*** + ***11f. (12%)***
other		Count (% per column)										**Sum for D3 (%)**	Count (%)			**Sum for D4 (%)**
undetermined	Varia	33 (31%)		16 (31%)	10 (7.5%)	4 (2%)		16 (15%)	21 (26%)	2 (1%)	55 (24%)	***156 (12%)***	8 (18%)	95 (17%)	10 (12%)	***113 (16%)***
plant imprints on mudbrick													20 (44%)			***20 (3%)***
charcoal, wood	Wood particles	18 (17%)	1 (33%)		2 (1.5%)	2 (1%)	80 (43%)	2 (2%)	1 (1%)		1 (< 1%)	***107 (8%)***	8 (18%)	330 (59%)		***338 (49%)***
**Grand Total**		**87 (100%)**	**3 (100%)**	**52 (100%)**	**134 (100%)**	**162 (100%)**	**187 (100%)**	**107 (100%)**	**82 (100%)**	**213 (100%)**	**226 (100%)**	**1273 (100%)**	**45 (100%)**	**564 (100%)**	**84 (100%)**	**693 (100%)**

[U #]—useful (food/ medicine/ fodder) REF #. Preservation marks: w - for whole; f - for fragments.

In the following we address four key issues: Construction of the plant database from the two consecutive temples, and the areas adjacent to them, including identification of all plant species (detailed list of sampled areas, contexts and loci see in Suppl. Table). The prevalence of local crops would suggest agriculture-oriented rites in the temples, while the dominance of wild plants shows the role of wild natural resources.

Comparative study of the temples’ plant ecology and biogeography, in relation to modern local flora of the site’s vicinity and of neighboring biogeographic regions. These data enable us to address the significance of different natural habitats, geographical distances, as well as vectors of connections with remote entities.

Analysis of the plant assemblages’ phenology to reconstruct possible seasonal/monthly dating of the collection and deposition of various plants in the temples. Although charred plant preservation from a burnt settlement is often limited to seeds and fruits, their unripe forms show the use of flowers and fresh vegetables in the temple.

Spatial reconstruction of plant-related activities in the temples and associated contexts, such as crop storage, processing, bedding, and fodder. This includes a study of the ethnobotany of the temple plants, known in neighboring archaeological cultures to be associated with medicine, symbolism, and other functions.

## Results and discussion

### Taxonomic composition, spatial settings, and density of finds

The plant assemblage represents 52 archaeological contexts and comprises ca. 2,000 plant macrofossils (Table 1, Supplemental Table). The identified specimens include 47 taxa: 26 species, 13 genera, and six family levels.

The density of plant findings is low in both Strata (0.1–6.0), yet plant finds were spread throughout (Table 1, Fig. 4a, b). The low density of plant finds, and their type of preservation through charring, suggest symbolic amounts of plants for offering and their burning inside the temple. It is also possible that the space was kept well-cleaned between the rites. Notably, in both strata, there is no evidence for communal storage of crops in silos/bins, as well as no evidence for intensive wood burning for bonfires.

Furthermore, the combination of staples, weeds, and chaff remains indicates that temple food did not arrive as clean, processed food—it was prepared in situ as a critical component of cultic practice. This corresponds to food serving vessels found in the Gath temples, as well as a clay oven (tabun), and grinding stones (in RW) within the temple compound (Fig. 3)^31^.

The Permanova test (one-way) used for testing the similarity between the two plant assemblages from D3 and D4 revealed that these are quite similar concerning their taxonomic composition (p-value is 0.17, total sum-of-squares SSt = 88.07, within group sum-of-squares SSw = 79.7, F is 1.258; Sørensen–Dice coefficient of similarity is 0.566).

Considering the spatial distribution of finds, the temples’ spaces differ in plant representation from the adjacent spaces (nMDS for taxa presence/absence data; left vs. right quadrants, Fig. 4a–b; d–e). This tendency holds for both temples, indicating that the inner rooms are ordinated closer to each other, despite being from different strata. Hence, the plant assemblages signify similar types of human activity in these temples.

Analysis of the spatial distribution of functionally different plant finds within Temple D3, revealed that the probability of finding food waste inside the temple is lower than expected under independent distribution, while wild plants are over-represented inside the temple (χ^2^-test; Table 2, Fig. 4b; d–e). Moreover, plants that are unrelated to disturbed and agricultural habitats make up more than half of the plant assemblages, suggesting their own significance in both temples (Table 1). Among these, seven species are well-known as fodder plants. The data suggests the presence of live herbivores in and around the temple compound.

**Table 2:** Correlation between plant functionality (food, chaff and weeds, and wild plants) and their relation within Temple D3 using the χ^2^ test.

Plant's spatial association	Food plants	Waste (chaff & weeds)	Wild plants
Food plants		χ^2^ (df = 6) = 102.78, P < 0.001	
Waste (chaff & weeds)	χ^2^ (df = 2) = 228, P < 0.001		χ^2^ (df = 2) = 336.74, P < 0.001
Wild plants	χ^2^ (df = 2) = 140.91, P < 0.001		

Diverse wild plants (growing in natural habitats), found next to the altar and in the back room among the votive vessels, suggest that they could have been used for bedding and for decorating the votive vessels. Also, among these plants, several possess a long history of ethnobotanical data (Table 1– useful plants). These useful plants include herbs, such as cheese weed (*Malva parviflora*), cleaver (*Galium* sp.), white goosefoot (*Chenopodium album*), etc. In addition, grains of a wild cereal—lovegrass (*Eragrostis minor*) (Fig. 5f), which was used during famines^32^—were the main plant component in a storage jar (D3, L 149515 B 1495041), along with remains of grape (*Vitis vinifera*), flax (*Linum usitatissimum*) and other plants. Another find in D3 was Syrian cephalaria (*Cephalaria syriaca*), known for its edible, oil-rich seeds and used as an anti-staling agent for bread dough^33,34^. It should be noted that this species was found beside the altar (L D15BW08). The diversity and spatial distribution of plant species suggest that the Philistines used a wide diversity of plants in their religious practices.

### Crops in the temples

Eleven food-related species, including cereals, pulses, fruits, oil plants, vegetables, and condiments, were found in the temples. Hulled and naked wheat (Fig. 5g), barley, olive, bitter vetch (Fig. 5k), fig, and grape were found in both temples and, unique for D3, flax and grass pea (Table 1). Grain staples represent the local diet and can be dry stored. Olives and linseed are edible and serve as sources for oil extraction^35^. Flax seeds in the temples (D3: L 149515, L 149817) may relate to the significance of the plant as a fiber source. The rich assemblage of loomweights found in the temple connects them to the local goddess Asherah. According to biblical texts, women weaved for Asherah in the Temple during the time of the Judean King Josiah (II Kings 23:7)^29,36^.

All except the oil plants can also be processed into alcoholic beverages, whose presence in the temples is well attested by ceramic vessels and microbiological analyses^37^. The crops comprise 21% of the plant assemblage in D4, and 68% in D3 (Table 1; Fig. 4d-e). The dominant food category in D4 is cereals, while in D3 cereals and pulses are found in similar quantities. Among the cereals in both temples, the complete and clean grains outnumber the broken grains (43 and 8, respectively), while cereal chaff is dominant (164 glumes and spike axes fragments). Chaff protects cereal grains from both pests and desiccation during storage, and the fine cleaning of grains of cereals usually is the last stage of preparation before milling or soaking and cooking. As for grapes—complete pips outnumber broken pips (48 and 18, respectively). The data indicate using raw, uncleaned crops and fruits inside the temples.

### Wild herbs for reconstructing food, medicine, and mystics in the temples

#### Chaste tree of Hera in the Philistine temple at Gath

The discovery of ca. 100 chaste tree fruits found in the D3 temple is unique in its abundance when compared to other sites in Israel (Fig. 5a, Table 3). The large quantity and its deposition among votives, unequivocally indicate the plant’s intentional use in the temple. This local shrub, growing on riverbanks, is known in contexts of food-related plants starting from the Lower Paleolithic (Table 3)^38^. The plant is used as a substitute for pepper, a moth repellant, and as a source of yellow color^39,40^. Chaste tree fruits were retrieved from the cargo of the Late Bronze Age Uluburun shipwreck (late fourteenth century BCE), showing that they were either traded or used, on board or at the ship’s destination^41^. Nicander (second century BCE) and Dioscorides refer to this plant as medicinal and as an aphrodisiac and abortifacient^15,42,43^. Clinical studies have confirmed its efficacy in treating several conditions, and the danger of using it during pregnancy^44^. Recent chemical studies have revealed its opioidergic, anti-inflammatory, antiproliferative, and other properties^45^.

**Table 3:** Lilac chaste tree, *Vitex agnus-castus*, in Bronze and Iron Ages of the southern Levant.

Site	Time	Context	Chaste tree component in total seed count (%)	Stratum, Locus	References
Ashkelon	604 BCE	Bldg. 58 room 58	1 out of ca. 29, 000 seeds (< 1%)	Grid 50 Phase 7	[^113^, Table 23.2]
Aphek	10th cent BCE	Grain storage pits	16 out of 12,120 (< 1%)	Stratum 8,L 4015; 4026 (1), 4813 (1), 5013 (3)	^114^
Aphek	11th cent BCE	Threshing area	4 out of 3980 (< 1%)	Stratum 9, L 3456; 3609	^114^
Aphek	13th cent BCE	Food storage rooms, Palace VI	1 out of 411 (< 1%)	L 1731	^114^
Gath	1310–1250 BCE	Large public bldg	1 out of 2205 (< 1%)	Stratum E4b, L E15AG03	^115^
Tel Beth Shemesh	14th cent BCE	Palace	1 out of 11,803 (< 1%)	LBA IIA palace, L 6062.04 crops in vessel	^116^
Tel Gezer	16th cent BCE	Midden	20 out of 3988 (< 1%)	Field VI (no data for sample size. Fruits number is “screened”)	^88^
Gath	25th cent BCE	Pebble installation with wood and dung ash	1 out of 916 (< 1%)	Stratum E5a, Room 104,108, Building 104,311 L. E15AT04	^117,118^

Although the chaste tree is widespread around the Mediterranean, its symbolic use is limited to ancient Greece. The plant is associated with cults in Sparta (Artemis and Asclepios), with Thesmophoria rites^15,46–48^, as well as with the cult of Hera on Samos^49,50^. Beyond textual evidence for the use of the plant for the adornment of Hera figurines and ritual sites, its fruits dominated the Heraion plant assemblage, which was rich in diverse fruits, crops, and wild plants^16,17,51^. Noteworthy, the Heraion of Samos is the only other temple/votive plant assemblage in which the fruits of a chaste tree are directly found, strengthening the claim that, also at Gath, it might be related to a goddess similar to Hera. The finds of chaste tree in the Philistine temple connect the plant’s long history in the Israeli archaeobotanical data with the plants’ medical and cultic use in the northeastern Mediterranean.

#### Crown daisy: food and rosette

Crown daisy (*Glebionis coronaria*, Fig. 5j) is an edible wild plant of the Asteraceae family, the young inflorescences and leaves of which, according to Dioscorides, were collected from the wild for use as green vegetables^15^. It was also valued for medicinal use and as an insecticide^52^. Dioscorides notes that garlands with crown daisies were used to crown statues of the goddess Artemis, and for personal protection from witches and magic^15^.

Note that the shape of the *Aster* family inflorescences resembles the well-known ‘rosette’ motif (Fig. 5i)^53^. This motif is widespread across the Late Bronze Age Mediterranean, and in Iron Age II Judah, Philistia, and Mesopotamia^54,55^. Although it is impossible to discern the origin of the motif and its connection with specific plant species, it is plausible to propose that the similarity in shape was obvious for the gatherers of crown daisy flowers.

#### Bitter vetch and Jerusalem

Bitter vetch (*Vicia ervilia*), according to Dioscorides and Pliny the Elder (first century CE), is a food, fodder, and medicinal plant (Fig. 5k)^15,56,57^. This annual low-growing plant is also associated with the Talmudic (second century CE) *borit karshina*—an ingredient listed in the incense offerings used in the Tabernacle and the First and Second Temples in Jerusalem^58^. This multipurpose plant, however, was found in the courtyard and inside the temple, so its exact use cannot be identified.

#### Poison darnel

Poison darnel (*Lolium temulentum*) is a weed found in wheat fields, and its poisonous properties (when added to flour) were most probably known to farmers. Yet, its grains were found in the western courtyard in Temple D3 and inside Temple D4 (Fig. 5h).

Poison darnel is a natural source of ergot fungi containing an LSD-type alkaloid, known to be hallucinogenic, used by midwives and as a fortifying ingredient in brewing^55,59,60^. Its use in cultic contexts as a psychoactive is mentioned in relation to the Greek Eleusinian Mysteries, associated with cults of agriculture^61,62^.

In sum, it is possible that the wild plants were part of the plant offerings as an integral component of the harvest (bitter vetch and poison darnel), yet chaste tree does not grow in crop fields, while crown daisy is easily discerned from the cereals. Thus, it is plausible that temple ritual praxis included the use of medicinal and mood-enhancing additions to regular foods. The presence of strainer spout jugs within the temples suggests the offering and consumption of various fermented foods and beverages in situ. This fits with the importance of the alcoholic beverages in Philistine culture Gath^36,63^, as well with the proven use of plant-related oils with hallucinogenic properties in ritual chalices, and incense altars found in Philistine temples^4,64–67^.

### Crops for offerings: agronomy and biogeographic provenance

The two assemblages contain ca. 200 weed seeds (Table 1). Most of these plants still grow in the Shephelah (Judean Foothills). The ecology of poison darnel and bristle-spike canary grass (*Phalaris paradoxa*), indicates intensive winter cultivation^68^; little lovegrass—use of irrigated soils (Fig. 5f).

Gromwell (*Buglossoides incrassata*) (Fig. 5d) is a weed of cereals and pulses(^69^:3.67) yet does not currently occur in the Shephelah and is uncommon even in the two neighboring regions where it does occur—the Judean Mountains and the Samarian Desert. The plant is common only in northern Transjordan and on Mount Hermon(^70^:260). Notably, the gromwell was found, along with crops and other weeds, on top of the pebbled surface just behind the altar, near the jar from the Jerusalem area, mentioned above. Thus, the current biogeography of this species indicates that the Philistines used non-local staples for offerings, from Judah and perhaps from other regions.

### Timing of plant harvest and deposition

The phenology of crops found in the temples is diverse, and their harvests span from the end of March (barley) till the end of October and into December (olive harvest for oil production)^71^, revealing a variety of times for offerings of freshly harvested crops (Fig. 4c). The earliest among the wild plants, used when green, are the crown daisy and cheese weed; they both flower from February to May and are present in both temples. These plants suggest the significance of natural events (i.e., the end of winter) for Philistine rites. The long-lasting scabiosa (a possible *immortelle*) suggests a year-round need for plant adornment of the temple (Fig. 5e).

Two unique plants, found only in D3, add significant data on the final phase of the sanctuary and settlement (Table 1, D3). The chaste tree flowers when most of the local plants are already at the fruiting stage, by the end of June (Fig. 4c). As its fruits in the assemblage are mature and abundant, it is likely they were gathered from July onward. Lovegrass matures from September to January (Fig. 4c). As its grains are found mixed with grape and flax, they apparently were harvested with these crops. Considering the differences in the harvest seasons of these two crops, the lovegrass was most probably collected with grapes (Fig. 4c). As fresh grapes are not traded over long distances, the time of their last deposition in the temple was likely during early autumn. In sum, the data indicate that the city of Gath was destroyed in the late summer/early autumn.

### Integrating comparative insights into the discourse of ritual food, symbolism of staple crops and agriculture

The food plants found in the temples are typical local staples^2,25,72,73^. The presence of charred finds of unprocessed crops and their weeds indicates the burning of samples of harvest products. Although the plant assemblage from a Philistine temple at Tell Qasile differs in quantity and plant list, these temples are alike in their diversity of feasting vessels and storage jars. This data supports the biblical description of the Philistines as worshippers of Dagon, an agriculture-related deity^22^. Also, a decorative motif—a faience ornament in the shape of a grape cluster—was found next to a cultic installation in the twelfth century BCE Philistine Ashkelon, reinforcing the importance of agriculture in the Philistine cult^74^.

The presence of staples in temples is also known in local Canaanite (e.g., Deir ‘Alla) and Israelite/Judahite cults (e.g., Shiloh, Moza), yet in contrast to the Philistines, those temples are known for communal food storage in silo and grain bins, along with storage jars^75–77^. In contrast, some cultures, beyond the southern Levant, show similarities to the Philistine pattern of plant use in their temples. Indeed, tableware, along with the burning of small portions of staple food, is known from Ur (mid-third millennium BCE), the ritual well at Ebla (MBA), the temple of Artemis/Apollo at Kalapodi (LB/IA), and from a later sanctuary of Kore and Demeter at the northern outskirt of Corinth (fifth century BCE)^78–81^. These data indicate possible northern and western influences on Philistine cultic practice.

#### Offering of traded goods

A non-local gromwell species in the temples indicates offerings of traded goods. This finding is supported by a non-local storage jar near the altar of the D3 temple and fits well with accumulated data on the economic relations of Philistia and Judah throughout the Iron Age^18,82–84^. The trade status of crops increased the value of the offering. At Tell Qasile the top component of the plant assemblage is pulses, identified as prestige imports already arriving in the Levant from the Aegean in the Middle and Late Bronze Ages^3,85–87^.

#### Flora and magic in the Philistine temples

**Figure 6:**
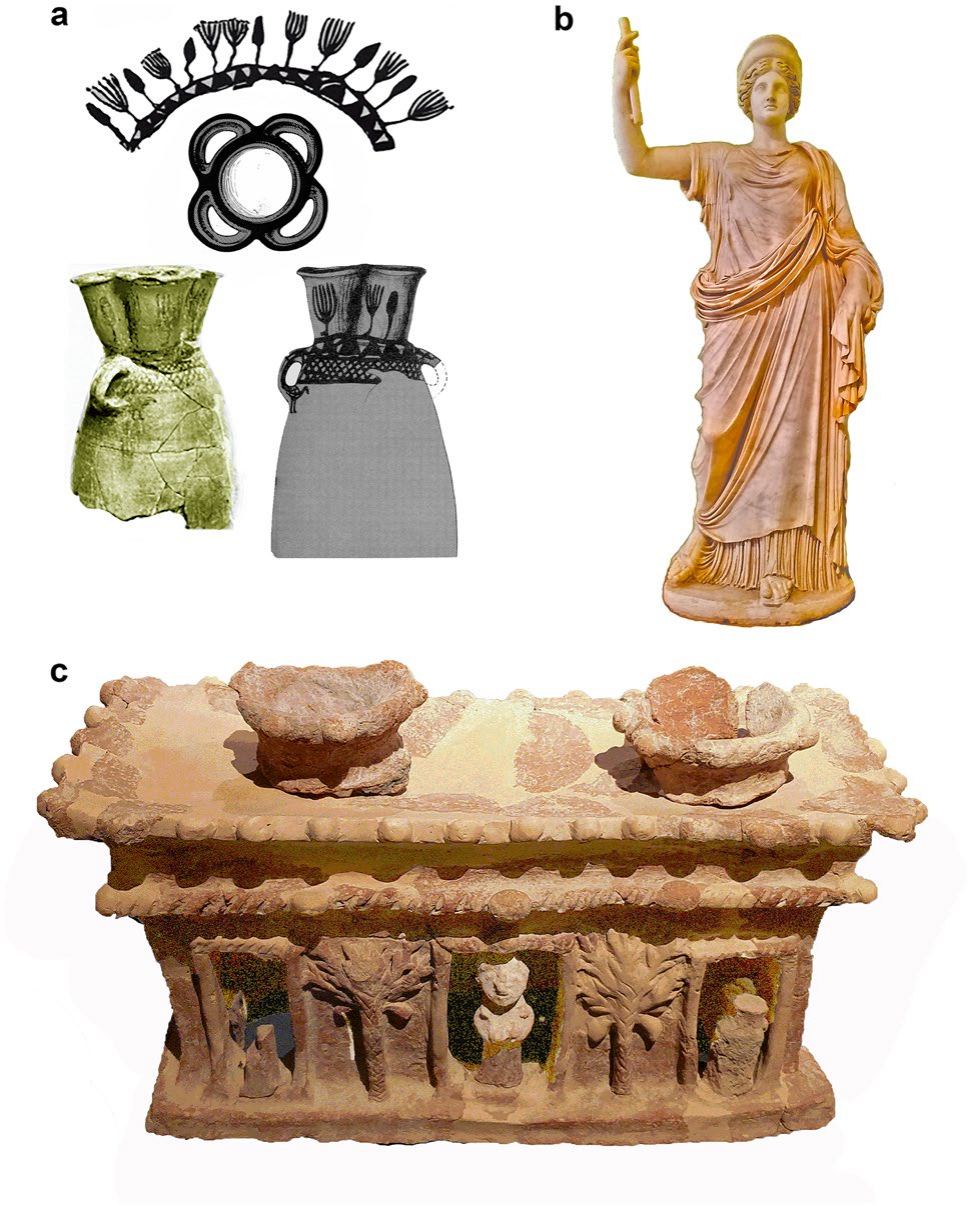
Philistines plant-related iconography. (**a**) Ceramic cultic jar, Tell Qasile, # 1302, Stratum X, Loci 142; 190: a frieze of plants painted on the upper part of the jar, flower-shape of the vessel, photo of the vessel, its drawing^72^. (**b**) Hera (Ephesus-Vienna type). Early first century CE copy of a Greek original (beginning of the fourth century BCE). Naples National Archaeological Museum, #6027. Photo by S. Frumin. (**c**) Ceramic Cult stand (CAT37), Yavneh. Muza—Eretz Israel Museum, IAA no. 2006–998. Photo by A. Maeir.

Wild plants found inside the temples, may indicate a cult of nature forces, or floral deity in the agricultural economy of Philistines at Gath. Although the use of plants in cultic contexts is common, local Canaanite symbolism is limited to crops and trees. The trees, potted plants, and garlands in sanctuary adornment, are known at many Levantine sites, including Ein Gedi (a large place for a tree at the temple entrance)^88^, Hazor (food offerings and a tree on figurine)^89^, Pella (date palm and food offerings)^90^, and Mari (date palm court)^14^, during the Bronze and Iron Ages. In the Philistine temple at Tell Qasile, there was a jar decorated with a presumed sacred tree/lotus (Fig. 6a)^91^. Philistine cultic stands from Yavneh and Nahal Patish include a date palm motif, while stands from Yavneh were also interpreted as ‘cultic flowerpots’ (Fig. 6c)^64,92^.

We surmise that finding wild plants with medicinal properties in the Philistine temples reflects their cultural differences from the Canaanites.

Floral symbolism is characteristic for instance for the Aegean goddess Hera, one of whose manifestations is as a floral deity (Fig. 6b)^93^. Mycenaean ritual offerings in Late Bronze tombs near Nemea (Argolis) also included diverse and numerous wild plants whose frequency exceeds field crops and fruits^94^. The cultic role of wild medicinal plants, some with psychoactive/toxic and decorative characteristics is known from various temples, such as Early Bronze Age Ebla^95^, and Late Minoan Crete^96^, and at the Phoenician sanctuary (ca. eighth/seventh centuries BCE) of Melqart/Herakles in Sicily^9^.

We assume that the presence of complete seeds of pulses (found in Gath and Tell Qasile) may be regarded as votives connecting to the magic of the plant’s ability to recover and propagate from an inert and toxic state, i.e., revivification. Indeed, pulse grains are stone-hard and toxic when dry^35^.

Nevertheless, the fact that the wild plants in the assemblages are widespread in the Shephelah may also show that the Philistines used any fresh plants that could be used for decoration. Kletter et al.^64,65^, when considering the large number of bowls and chalices among the offerings in tenth-eighth centuries BCE Yavneh, already proposed the use of locally available plants for the Philistine ritual^65^.

#### Spring, Sun, and the circle of rites

The crown daisy, found in both temples and flowering from February to May, indicates the importance of spring in their cultic practice (Fig. 4c). Regarding the offerings of freshly harvested crops, at least five more dates can be proposed until the end of the olive harvest in December. That said, all local crops are dry-stored and traded, so their offerings may have occurred throughout the year.

Plant-related decorations in the temples probably included freshly gathered leaves of the chaste tree, mallow and grape, branches of the olive tree, grape clusters, and cereal sheaves. Also, crown daisies, silvery scabious, and chaste tree have relatively large, flamboyant, and brightly colored flowering heads (Fig. 5b, i).

The temples seem to be oriented along NNW-SSE and ENE azimuths (Fig. 3), like those at Tell Qasile and the Heraion of Samos^50,97^. Although the orientation may follow the landscape, these directions correspond with the mid-summer celestial events (see Supplement), associated with fertility rites and the temporality of human eminence. Mid-summer symbols are wildflowers, bonfires, music, dancing, and symbolic purification in open water sources such as rivers or lakes^98^.

#### The chaste tree, lovegrass, and dating of the temples’ final use

Chaste tree fruits are the most abundant plant component of the temples. In Greco-Roman sources, this multi-purpose plant is related to independent female deities, namely Artemis, Hera, and Demeter. Their widespread cults were related to the magic of childbirth and death, of fertility and plenty. These female deities accord with the wide occurrence of female figurines in Philistine contexts^99^. These figurines connect Philistines with widespread cults of the Aegean or Mycenaean Great Mother Goddess^100–102^.

The chaste tree also relates to the Asclepios cult in Sparta. Of note, cultic paraphernalia at Gath includes phallic-shaped *situlae* also found at Philistine Ashkelon, as well as in the Asclepios sanctuary at Sparta^103^. These findings further connect the Philistines with the symbolism of revivification. Additionally, fresh water is significant to all the cults mentioned above, hinting at a possible connection between the rituals at the temples at Gath and the nearby river [e.g.^104^]. The temples at Tell Qasile and Sparta are also located near rivers.

The plant-specific connections of the temple at Gath with cults from the regions of Argolis, Sparta, and Samos, provide a new framework for understanding the complex origins and cultural connectivity of the early Iron Age Philistines. Geographically, the data connect the Philistines with sites situated on 37°N latitude (Fig. 1a). Also, the ancient Argolid was proposed as the origin for the Hera and Artemis cults, as derivative developments from a Bronze Age Mycenae Potnia^49^.

Finally, the phenology of the daisy suggests early spring as an important date for rites, while both the chaste tree and lovegrass strongly indicate the end of summer/early autumn as the period for the final offerings at the temple, and thus the city destruction by Hazael of Aram (ca. 830 BCE).

## Conclusion

The present study presents plant data from two Philistine temples, offering novel insights and understanding of Philistine ritual practice during the Iron Age I/II and IIA (ca. 1000–830 BCE). Our results indicate the significance of the spring, wild plants and agriculture, trade, natural cycles, and fresh water. This new data indicates knowledgeable activity by temple personnel regarding the use of plants with mood-affecting features. We expect our method of quantitative and qualitative analysis of total plant assemblage to be highly relevant for analyzing other ancient cults and for the study of the cultural and cultic history of the region and beyond.

Evidence of the chaste tree, crown daisy, scabious, and poison darnel in temples fills lacunae in the cultural history of these plants, the history of the rosette motif, and reveals Philistine connections with the cults of Hera-Artemis-Demeter-Asclepios. The Philistine temples supply the first-known case of the connection of crown daisy and scabious to cultic depositions, and the earliest example of the chaste tree’s cultic use. The textual sources for the connection between the Hera cult and the chaste tree appeared later than the Philistine's temples, so no absolute connection can be made between the two. Yet, the uniqueness of the chaste tree in ritual contexts in both Philistia and Samos, and the clear connections between Philistine culture and Aegean traditions, are worth noting. We propose to study further possible parallels in the cults of the Philistines and rites associated with the cult of Hera. Significantly, additional possible connections can be seen in the many loom weights found in the temples at Gath, which are known from cult locations connected to Hera in the Aegean as well^105–107^. Connections between Aegean and Philistine cults have been noted before^108,109^, but here we propose the first possibility for such a connection related to plant use in cultic contexts.

## Materials and methods

### Seeds and fruits sampling design and plant assemblage database construction

To obtain the relevant archaeobotanical data the temples and their surroundings were carefully sampled for macrobotanical plant remains during the excavations. A breakdown of the strata, sampled structures and loci, sediment volume (V), numbers of plant finds (N), and plant densities (N:V) are given in Table 1, Fig. 3, and Suppl. Table. The samples originated from well-defined and sealed contexts, such as an altar, and votive assemblages including many ceramic vessels, loom weights, grinding stones, as well as a shell of *Tonna galea*, dated to tenth-ninth centuries BCE (based on ceramic chrono-typological analysis and ^14^C dating). The plant assemblage discussed here was extracted from ca. 1,100 L of sediment samples (rich with calcareous fragments, silty quartz [up to 0.5 mm], limestone, chalk, and *nari* [calcrete] inclusions), and from ca. 40 vessels. The 348 samples were collected during the 2008–2015 seasons of excavations.

Large samples of sediments were floated in a field laboratory using the A-FLOAT system, while smaller samples were dry-sieved using sets of geological sieves. After drying in the laboratory, the washed samples were also sieved, and all plant finds larger than 0.3 mm were collected and studied. This allowed us to build a comprehensive database of plants, including macro- and microscopically observed seeds and fruits. Due to the fragility of the plant finds, further cleaning was done with extremely soft brushes, as repeated watering dissolves the charred macro remains. Seed and grain fragments exceeding half of the complete size were counted as a complete sample.

Plant finds were studied under a magnification of up to × 63, and selected specimens were documented using an Olympus SZX10 imaging system (DP73). Detailed descriptions of the extraction and identification methods are published elsewhere^110^. All specimens discussed in the present work were deposited in the Archaeobotanical Collections of Bar-Ilan University, Ramat Gan (Israel). Plant species identification was tested in comparison with recent material found in ‘Israel Natural Nature Collection of Seeds and Fruits’ hosted in the Archaeobotany Laboratory at the Martin (Szusz) Department of Land of Israel Studies and Archaeology, Bar-Ilan University, and in the National Herbarium (HUJI, Jerusalem).

Wild plants and crops nomenclature, species distribution, species phenology in the southern Levant (time of flowering), adaptation to agricultural plots, and crop weeds, are based on the published datasets of *Flora Palaestina* and our survey of the immediate environment of the site^35,69,70,111^.

Data on species in ancient ethnobotany follow textual sources, such as those of Nicander (a Greek poet, physician, and grammarian, second century BCE) and Dioscorides (a Greek physician in the Roman army, 50–70 CE), who recorded multipurpose use of crops and wild plants in the ancient economy of the eastern Mediterranean^15,42^.

We addressed the location and orientation of the temple precinct within the settlement and its immediate surroundings. Although the orientation may follow the river course and its terraces, it affects the view of the temple visitors and its personnel. As the temple is situated inside the city and by its wall, most probably, its orientation affected the sky view. Movements of celestial objects were used for time measurement, including the timing of sowing and harvest.

### Plant assemblage analyses. Statistics

The plant assemblages from the two temples discussed here might have differences due to the following reasons: 1. Deposition circumstances—the temples have different termination circumstances (reconstruction vs abandonment); 2. Spatial—different areas were sampled within the two temple precincts (Table 1, Fig. 3). Considering the possible deposition variation, we compared the two plant assemblages (D3 vs D4) using the one-way PERMANOVA test, using Sørensen–Dice coefficient of similarity (PAST software^112^). To address the possible spatial variation in plant diversity across the temple precinct we applied non-metric MDS analysis (k = 2, number of dimensions), using Bray–Curtis similarity index, to ordinate sampled spaces/rooms (taxa presence/ absence, Fig. 4A). This statistic accounts for as much of the variability in the data as possible and transforms possibly correlated variables into a smaller number of uncorrelated variables (principal components).

Then, we compared the plant list and species frequencies in the various spatial contexts of the temples. We tested the spatial spread of different useful plants, like cereal, pulses, fruits, etc. in different spaces of the temples (Fig. 4c). This included the temples' inner spaces (rooms and courtyards) and spaces next to them, which might have had cultic functions (Fig. 4b, d).

As for plant use in temples, we addressed the question of the spatial spread of functionally different plant finds within the Temple D3: food (cereal grains, pulses, and fruits) vs. food waste (chaff and weeds) vs. wild plants (plants growing in natural undisturbed habitats only) using the χ^2^-test (Table 2). Indeed, a temple precinct’s inventory may contain raw offerings of harvest (grain and fruits), components of prepared meals (e.g., bread and wine), waste from the temple food (chaff, weeds), as well as branches, inflorescences, and fruits used for adornments and bedding, as well for symbolic burning and bonfires. The null hypothesis was that there are no differences in the plant groups' deposition within the temple components: inside the temple rooms, in courtyards connected to the temple, and in built spaces outside the temple unconnected to its inner rooms. In this case, we expected that the test statistic computed from the observations would follow a χ2 frequency distribution—random distribution (Fig. 4d). Observed frequency (F obs) for each functional group is the actual number of plant finds associated with a group *I* in a temple area *j*. Expected frequency (F exp) is calculated using the total number of plant finds associated with a specific functional group, the total number of plant finds of all types in each temple area, and the total number of plant finds:

$${\text{F}}\;{\text{obs}}(i) = Nij\quad F \exp(i) = \frac{NNi \times NNj}{{NN}}$$

N*ij*—number of plant finds associated with *i*-functional group in *j*-area; N*j*—number of plant finds retrieved in *j*-area of the temple; NN*i*—total number of plant finds associated with *i*-functional group; NN*j*—total number of plant finds in *j*-area of the temple; NN—total number of plant finds in the temple.

Plant phenology data (the range of harvest time for each species) was used to address the possible time of plant harvest and deposition in the temple, in case of the use of fresh leaves/flowers or first fruits (Fig. 4c). Indeed, Tell eṣ-Ṣâfī/Gath is situated in the Mediterranean climate, and throughout the calendar year — some plants are in flower, while others are in harvest, or seasonally dormant or germinate^111^. Thus, analysis of the species phenology was applied to address the possible variation in plant offering times, and by this—seasonality of rites.

We discussed the new data with what is already known about the Philistine diet, as well all plant-related information from Philistine cultic paraphernalia, such as incense altars and ritual vessels, cult stands, etc. In addition, we correlated the Philistine temples’ plant use with the local Canaanite, Israelite, and Judahite cultures, as well as with other ancient Near Eastern and Eastern Mediterranean traditions (Fig. 1). This includes as corresponding archaeological data, as well as textual sources, such as Biblical and Talmudic texts, the Greek epic poem Iliad, Samian texts, sources for Thesmophoria and Eleusinian Mysteries, etc.

The comparative qualitative and quantitative approach to the archaeobotanical data enables to address relationships between the Philistine economy and the cultic rites, unravel the seasonal timing of these rites, discuss the temples’ decoration with plants, and aid in better understanding the cultural trajectories and continuities both within the Philistine culture and other Levantine cultures, and in the eastern Mediterranean in general.

### Supplementary Information

Supplementary Information.

## Data Availability

The data that support the findings of this study are included in this published article (and its Supplementary Information files).
