# Supplementary material for: Plant-related Philistine ritual practices at biblical Gath
Source: Sci Rep. 2024 Feb 12;14:3513. doi: 10.1038/s41598-024-52974-9 (PMC10861565; doi:10.1038/s41598-024-52974-9)

## Plant-Related Philistine Ritual Practices at Biblical *Gath*

Suembikya Frumin*^1,2^, Aren M. Maeir^2^, Maria Eniukhina^2^, Amit Dagan^2^, Ehud Weiss*^1,2^

Corresponding authors: [Suembikya.Frumin@biu.ac.il](mailto:Suembikya.Frumin@biu.ac.ill); [Ehud.Weiss@biu.ac.il](mailto:Ehud.Weiss@biu.ac.il)

1. Archaeobotany Lab, The Martin (Szusz) Department of Land of Israel Studies and Archaeology, Bar-Ilan University, Ramat-Gan, Israel.
2. The Institute of Archaeology, Bar-Ilan University; The Martin (Szusz) Department of Land of Israel Studies and Archaeology, Bar-Ilan University, Ramat-Gan, Israel.

**Supplemental**

Supplemental table

| Strata | BLDG/ROOM/COURT | Locus | Basket | Context |
| --- | --- | --- | --- | --- |
|  | BE: BUILD. 16D04D02 | 16D04D07 | 16D04D080 | pillar base |
|  |  | 16D04D07 | 16D04D117 | pillar base |
|  |  | D15BG11 | D15BG100 | juglet |
|  |  | D15BQ06 | 16D04C024 | juglet |
|  |  | 16D04D02 | 16D04D021 | vessel |
|  |  |  | 16D04D022 | jar |
|  |  |  | 16D04D060 | by vessels |
|  |  |  | 16D04D079 | by vessels |
|  |  |  | 16D04D088 | by vessels |
|  |  |  | 16D04D100 | by vessels |
|  |  |  | 16D04D101 | by vessels |
|  |  |  | 16D04D104 | by vessels |
|  |  |  | 16D04D105 | by vessels |
|  |  |  | 16D04D108 | by vessels |
|  |  |  | 16D04D125 | by vessels |
|  |  |  | 16D04D126 | storage jar |
|  | BS: BUILD. D15BV04 | 17D13B03 | 17D13B020 | by skeleton D15BV04 |
|  |  | D15BV04 | D15BU038 | by skeleton D15BV04 |
|  |  | D15BV06 | D15BV050 | by skeleton D15BV04 |
|  |  | 149515 | 1495042 | storage jar |
|  | BSE: BUILD. 16D04D05 | 16D04D05 | 16D04D123 | storage jar |
|  |  | 16D04D05 | 16D04D124 | vessel |
|  | CN: COURT. D15AP04 | 17D94C07 | 17D94C039 | floor |
|  |  | 18D03B03 | 18D03B019 | floor |
|  |  | D15AP04 | D15AP040 | juglet |
|  |  |  | D15AP044 | vessel |
|  | CW: COURT. 119808 | 119808 | 1198025 | jug |
|  |  |  | 1198064 | juglet |
|  |  |  | 1198066 | juglet |
|  |  | 139906 | 1399027 | by vessels and loomweights |
|  |  |  | 1399039 | juglet |
|  |  |  | 1399087 | juglet |
|  |  |  | 1399100 | bowl |
|  |  | 149711 | 1497057 | skeleton |
|  |  |  | 1497084 | storage jar |
|  |  | 17D12A03 | 17D12A024 | Holemouth vessel |
|  |  | 18D03C02 | 18D03C018 | by vessels |
|  |  |  | 18D03C023 | tabun |
|  |  | D15BS02 | D15BS031 | floor |
|  |  |  | D15BS032 | vessel |
|  |  |  | D15BS033 | floor |
|  |  |  | D15BS034 | juglet |
|  |  | D15BT02 | D15BT02 | tabun |
|  |  | D15BU05 | D15BU016 | storage jar |
|  |  |  | D15BU024 | crater |
|  |  |  | D15BU025 | storage jar |
|  |  | D15BU06 | D15BU013 | floor |
|  |  |  | D15BU015 | floor |
|  | RW: ROOM 119610 | 119610 | 1196073 | under grinding stone |
|  |  | 119808 | 1198006 | floor |
|  |  | 149607 | 1496019 | chalice |
|  |  |  | 1496020 | jug |
|  |  | D15BL11 | D15BL009 | jug |
|  |  |  | D15BL010 | juglet |
|  |  | D15BL12 | D15BL036 | by vessels |
|  |  |  | D15BL037 |  |
|  | TC: ROOM 149807 | 149808 | 1498033 | by altar |
|  |  | 149810 | 1498040 |  |
|  |  |  | 1498047 |  |
|  |  | 149109 | 1491023 | next to altar |
|  |  | 149817 | 1498101 | behind altar |
|  |  | D15BW08 | 16D03D012 |  |
|  | Altar ROOM 149702 | 139508 | 1395419 | storage jar |
|  |  | 139610 | 1396067 | under barrel |
|  |  | 149702 | 1497070 | floor |
|  |  | 149709 | 1497050 | pavement |
|  |  | 149709 | 1497051 | bulk |
|  |  | [149709](javascript:void(0)) | 1497050 | by votive assemblage |
|  |  | 149712 | 1497123 | storage jar |
|  |  | 149712 | 1498010 | pavement |
|  |  | 149712 | 1498013 | pavement |
|  | TC: ROOM 149814 | 149813 | 1498057 | by votive assemblage |
|  |  | 149814 | 1498068 |  |
|  | TE: ROOM D15BQ08 | 149906 | 1499033 | floor |
|  |  |  | 1499036 | storage jar |
|  |  |  | 1499040 | storage jar |
|  |  |  | 1499061 | cooking pot |
|  |  |  | 1499064 | storage jar |
|  |  |  | 1499066 | by vessels |
|  |  | D15BG08 | D15BG102 | cooking pot |
|  |  |  | D15BG104 | juglet |
|  |  |  | D15BG106 | storage jar |
|  |  |  | D15BG108 | storage jar |
|  |  |  | D15BG109 | jug |
|  |  | D15BQ05 | D15BQ071 | vessel |
|  |  |  | D15BQ107 | lamp |
|  |  | D15BQ07 | D15BQ064 | storage jar |
|  |  |  | D15BQ069 | vessel |
|  |  | D15BQ08 | D15BQ081 | loomweights |
|  |  |  | D15BQ197 | jar |
|  |  |  | D15BQ206 | jar |
| D4 | CS: COURT. D15BP13 | 17D04C02 | 17D04C019 | jar |
|  |  |  | 16D04C005 | by tabun |
|  |  |  | 16D04C006 |  |
|  |  | 16D04C03 | 17D04C053 |  |
|  |  |  | 17D04C054 |  |
|  |  |  | 17D04C147 |  |
|  |  |  | 18D04C009 |  |
|  |  |  | 18D04C010 | tabun |
|  | TC: ROOM D15AN07 | 18D03A04 | 18D03A022 | floor |
|  | TE: ROOM 16D93D05 | 16D93D03 | 16D93D019 | by votive assemblage |
|  |  |  | 16D93D022 |  |
|  |  |  | 16D93D023 |  |
|  |  | 16D93D04 | 16D93D039 | by vessels |
|  |  |  | 16D93D040 |  |
|  |  |  | 16D93D044 |  |
|  |  |  | 16D93D045 |  |
|  |  |  | 16D93D046 |  |
|  |  |  | 16D93D047 |  |
|  |  |  | 16D93D048 |  |
|  |  | 17D94C03 | 17D94C023 | floor |
|  |  |  | 17D94C024 | floor |
|  |  |  |  | jug |

S1. Azimuth in the Iron Age in Tell es-Safi/Gath Area D, produced using Stellarium 0.19.1 (Georg Zotti, Alexander Wolf).


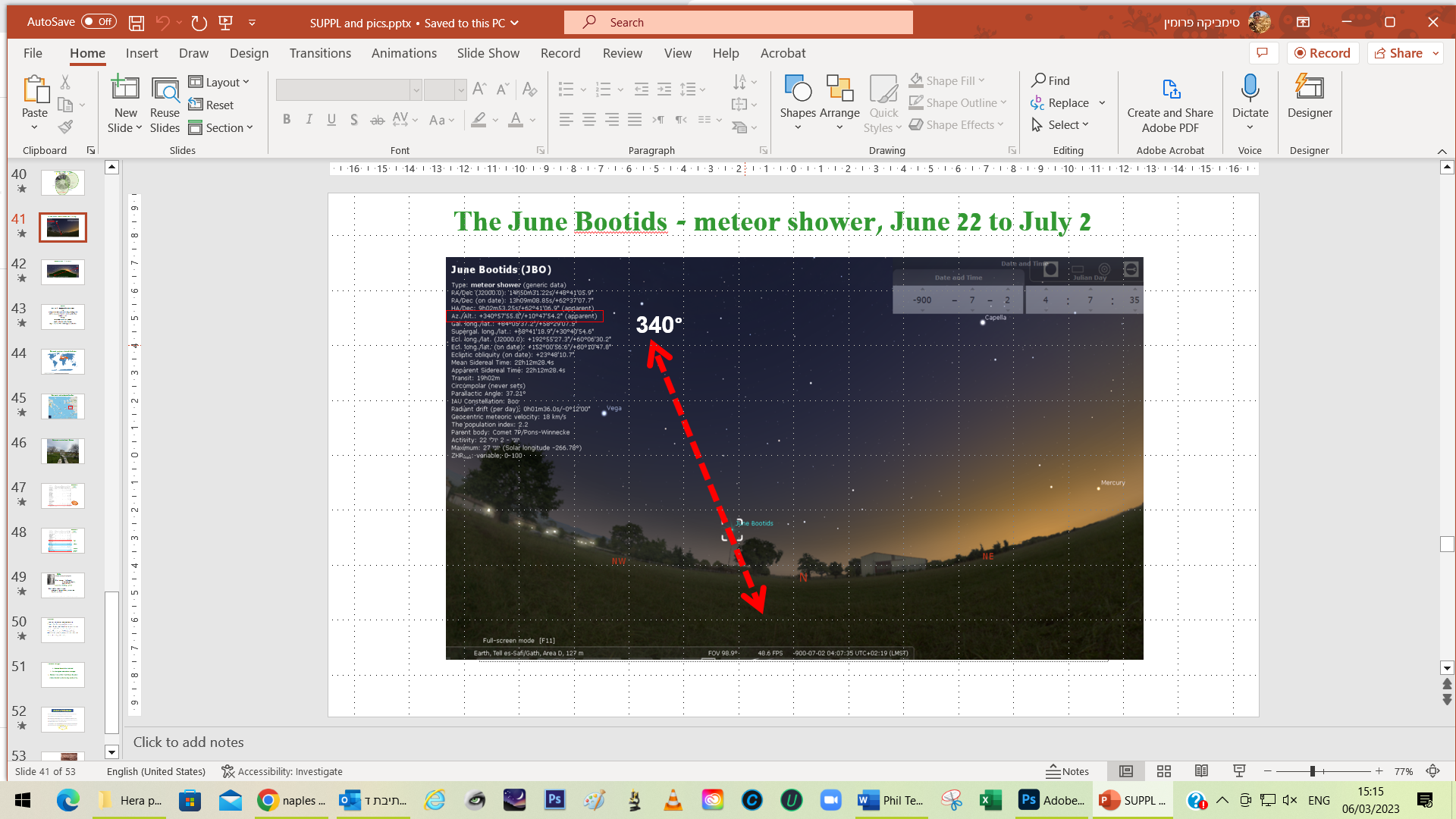

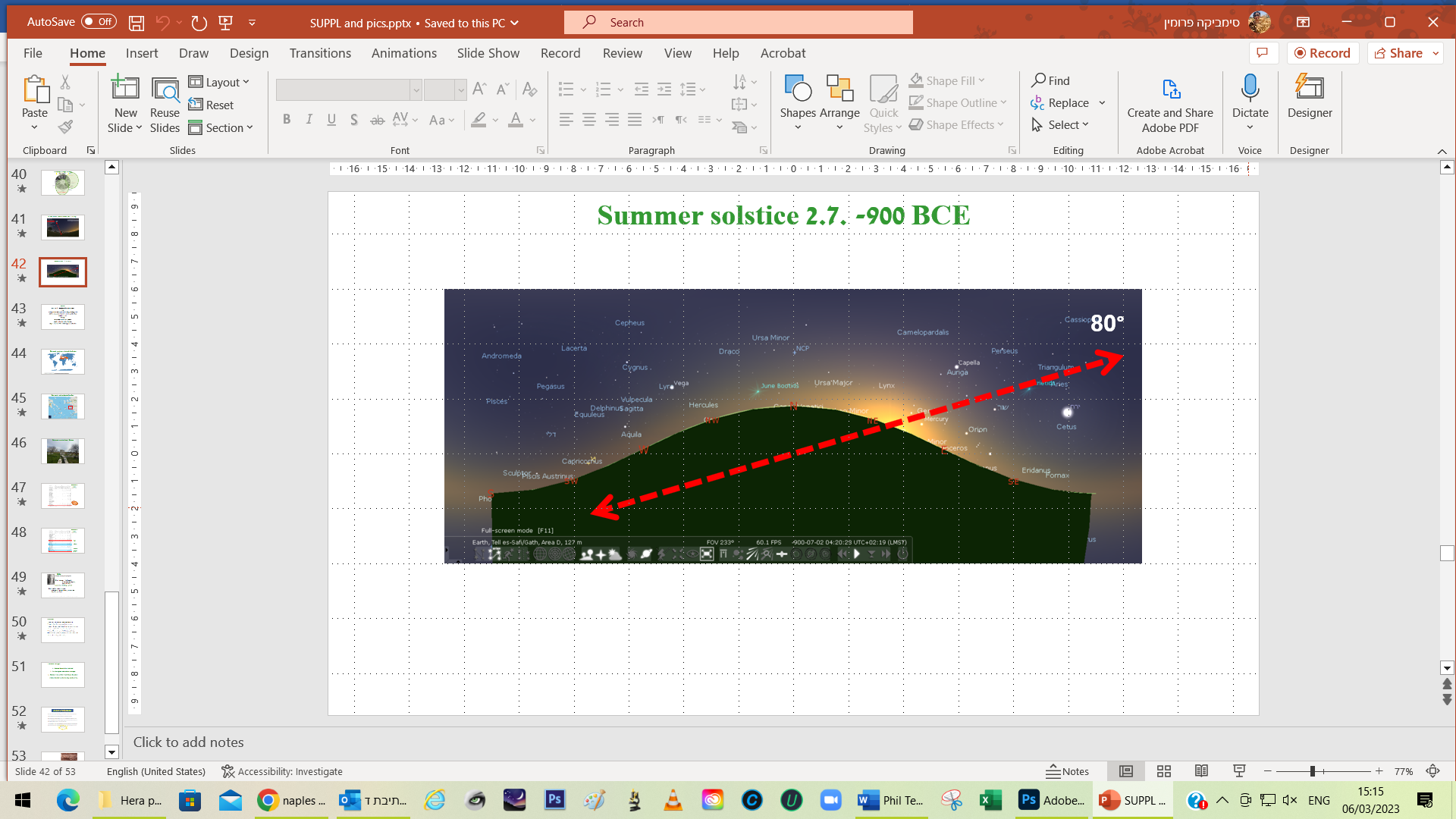

Supplement: Supplementary file 1 — Supplementary Information. [file 41598_2024_52974_MOESM1_ESM.docx]
